# Stress and coping experience in Nurse Residency Programs for new graduate nurses: A qualitative meta-synthesis

**DOI:** 10.3389/fpubh.2022.979626

**Published:** 2022-09-20

**Authors:** Peng Han, Xia Duan, Lingmin Wang, Xiaoping Zhu, Jinxia Jiang

**Affiliations:** ^1^Emergency Department, Shanghai Tenth People's Hospital, School of Medicine, Tongji University, Shanghai, China; ^2^Nursing Department, Shanghai First Maternity and Infant Hospital, School of Medicine, Tongji University, Shanghai, China; ^3^Nurisng Department, Shanghai Tenth People's Hospital, School of Medicine, Tongji University Shanghai, Shanghai, China

**Keywords:** new nurses, Nurse Residency Programs, stress, meta-synthesis, qualitative systematic review

## Abstract

**Objectives:**

To determine the stress experience and coping styles of new nurses during Nurse Residency Programs (NRPs) by identifying, appraising, and synthesizing data from the qualitative studies.

**Design:**

A systematic review and meta-synthesis of qualitative studies.

**Review methods:**

Eleven databases were systematically searched for relevant publications in March 2022. All qualitative and mixed-method studies in English and Chinese that explored the stress and coping experience during NRPs of new graduate nurses were included. The qualitative meta-synthesis was performed following the Preferred Reporting Items for Systematic Reviews and Meta-Analyses (PRISMA) recommendations. Two independent reviewers selected the studies and assessed the quality of each study. Meta-synthesis was performed to integrate the results.

**Results:**

A total of 13 studies revealed 13 sub-themes and three descriptive themes: multi-dimensional stressors, somatic and emotional responses, coping resources and coping methods.

**Conclusion:**

New nurses faced a lot of physical and emotional stress during NRPs, which had a negative impact on their physical and mental health. NRPs are a critical period for the career growth of new nurses. Effective management strategies must be implemented to improve nurse capacity, meet their needs, improve self-efficacy, and build organizational support, as this can improve the quality of clinical nursing and keep the enthusiasm and stability of the nursing team.

## Introduction

According to World Health Organization (WHO) predictions based on current trends, the global nursing workforce will experience shortages of 5.7 million by 2030. New graduate nurses leaving their positions shortly after they start work for various reasons is an important factor causing the shortage of nurses. To address the shortages predicted to occur by 2030, the total number of nurse graduates would need to increase by 8% per year on average, alongside an improved capacity to employ and retain these graduates (1).

In the early twenty-first century, the Institute of Medicine (2) published a seminal report, “The future of nursing: leading change, advancing health.” The report advocated that nursing students undergo Nurse Residency Programs (NRPs) to become nurses to establish a professional identity and improve the quality of nursing. Since then, NRPs have received great attention. Each country has relevant regulations for the NRPs, and the training cycle and workplace vary greatly. For example, China's Ministry of Health has issued regulations requiring new nurses in hospitals to undergo 2 years of training in four departments, i.e., internal medicine, surgery, critical care, and emergency (3). In the United States, NRPs last for 12 months and are conducted primarily in a designated department (4). The cycle of NRPs in the UK is 6–12 months, where nurses circulate between multiple clinical units according to a defined individual plan (5). Australia, Scotland, and Japan have similar content, and in these countries, the whole program takes 1 year to complete (6–8). Although the program, mode, and cycle of NRPs vary from place to place due to different cultures and medical conditions, NRPs is widely regarded as essential and useful for nurses' career, for the new graduate nurses to master the necessary work skills and quickly adapt to clinical nursing work, as well as to protect nurses from injury (9).

New graduate nurses were defined as nurses who worked within 2 years after graduation (10). From students to work, new nurses face the change of environment and their own role. It is reported that the turnover rate of new nurses in 1 year (turnover and job change) is 35–60% (10). New graduate nurses' experience of early practice represents a significant stage of building confidence and professional identity (11). The development of confidence and identity is essential for persistence in the nursing profession. Some new nurses experience the transition period of NRPs as a time of personal growth and fulfillment. A comparative study demonstrated that nurses' theoretical scores, clinical competence and post-competency had improved significantly compared with those before NRPs (12). For others, this is a period of stress. In addition to stressors that are obvious for the whole nursing population, such as high workload, staff shortages, and lack of emotional support (13), new graduate nurses are facing specific challenges associated with adapting to a new professional role, a phenomenon also known as a transition shock (14). These transitional stressors are derived from personal, interpersonal, and organizational factors (15). When new nurses suffer from adverse physical and psychological effects caused by transition shock pressure, it may lead to slow role transition, low job satisfaction, and increased turnover intention (16). More seriously, it can cause post-traumatic stress disorder in the long term (14).

Coping styles, also known as coping strategies, refer to reactions that change one's thinking and actions to maintain psychological balance when dealing with pressure (17). They are influenced by the individual's cognitive evaluation, personality, and physical and mental experience. In 2000, Anderson divided coping styles into two main categories, i.e., positive and negative (18). These two dimensions are independent and have different coping outcomes. Coping positively with stressors is often seen as difficult but necessary. During the NRPs stage, if new nurses can adapt well to the complex clinical environment and situation, this will undoubtedly benefit their whole career. Implementing strategies to overcome stressors according to the needs of new nurses is a concern shared by researchers and nursing managers, which is worth further exploring.

Our review aims to identify the embodiment and causes of physical and mental stress faced by new nurses during NRPs, and how it affects their health. At the same time, what factors affect their ability to thinking and behavior to cope with stress needs to be clarified. Few previous studies or systematic reviews have comprehensively reviewed the stress and coping experience in NRPs for new graduate nurses. This article highlights the relevance of the human factor in explaining nurses' physical and mental experience of career transitions. For example, communication with leaders or tutors, learning from each other among colleagues, etc. Meanwhile, we emphasized that coping with difficulties and stress and eliminating negative emotions is a process change, which will help managers to explore how to establish strategies from the practice of NRPs. Our review would determine the stress experience and coping styles of new nurses during NRPs by identifying, appraising, and synthesizing data from the qualitative studies. Identify new integrate themes and discuss its significance in nursing practice and the career growth of nurses.

## Methods

### Design

This exploratory qualitative meta-synthesis aimed to determine the stress experience and coping styles of new nurses during Nurse Residency Programs by identifying, appraising, and synthesizing data from the qualitative studies. This review enabled us to make recommendations to improve nurse retention rate and clinical nursing effect. The Preferred Reporting Items for Systematic Reviews and Meta-Analysis (PRISMA) (19) was used as a basis for reporting the review. A meta-synthesis approach was used to combine and present the qualitative findings (20). Inspired by Sandelowski et al. (21), meta-synthesis of qualitative research is based on the premise of understanding its philosophical thoughts and methodology, repeatedly reading the included literature and extracting the themes and hidden meanings so as to conduct inductive analysis, form new categories, and finally integrate new results. By synthesizing new results, a more profound and substantial explanation can be given to specific phenomena, creating new perspectives and so-called “third-level” findings, providing a more influential and persuasive final conclusion. Relevant articles were searched, and data were extracted and critically evaluated using a thematic synthesis based on the three steps outlined by Thomas and Harden (22), i.e., text coding line by line, developing descriptive themes, and generating analytical themes. This study met the requirements of the Helsinki Declaration.

### Search methods

**Figure 1:**
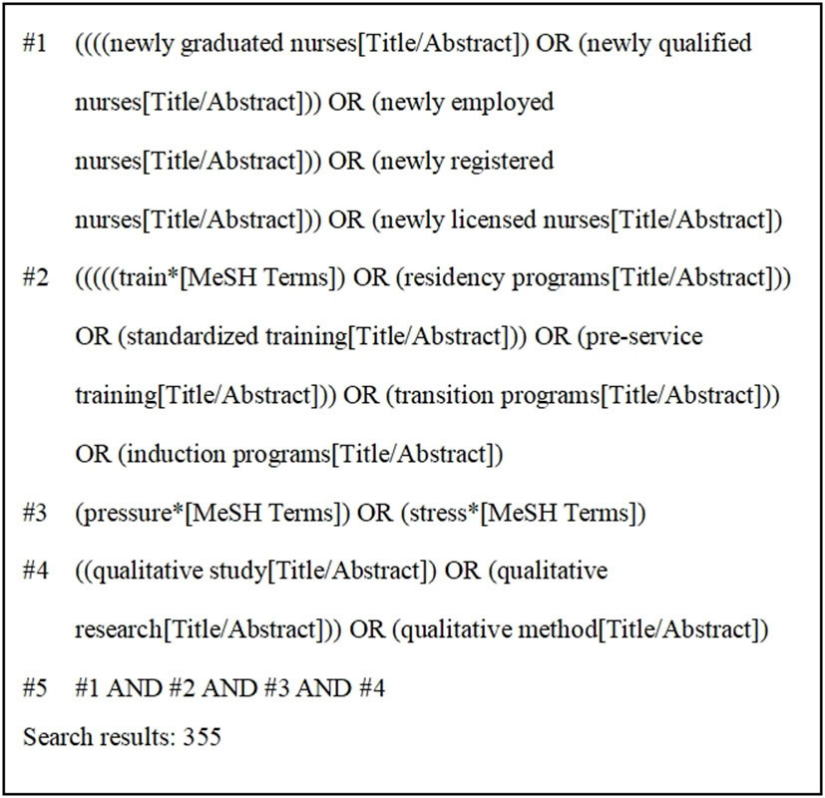
Search strategy in PubMed.

To ensure adequate performance in searches (i.e., recall, precision, and number needed to read), we selected a combination of 11 databases for the literature search of systematic reviews, including both Chinese and English databases that are widely used in the field of health care. Qualitative studies published in PubMed, Cochrane Library, CINAHL, Web of Science, Embase, Ovid, Elsevier, and Chinese databases, including Chinese National Knowledge Infrastructure (CNKI), Wanfang Database (CECDB), VIP Database, and China Biomedical Database (CBM) from the establishment of these databases to March 2022 were searched by two researchers (PH and LW) in April 2022. The search terms were developed, and subject headings were used where possible and adjusted for different databases. Four groups of keywords or MeSH terms were included and combined using Boolean operators: (1) newly graduated nurses, newly qualified nurses, newly employed nurses, newly registered nurses, newly licensed nurses; (2) train^*^, residency programs, standardized training, pre-service training, transition programs, induction programs; (3) pressure^*^, stress^*^; (4) qualitative study, qualitative research, qualitative method. To determine the eligibility of the potentially relevant studies, all titles and abstracts were reviewed by researchers. Figure 1 showed the search strategy with PubMed as an example.

### Inclusion and exclusion criteria

#### Study design (S)

The qualitative research or mixed-method studies from which qualitative data could be extracted, the primary qualitative research studies were included but were not limited to methodologies, such as phenomenology, grounded theory, action research, ethnography, and feminist research.

#### Participant (P)

Newly graduated nurses who were starting clinical work.

#### Interest of phenomena (I)

The real experience of new nurses in experiencing and adjusting to stressful events. The focus was on their stressors and coping styles in NRPs.

#### Context (Co)

Nurses who completed or were undergoing NRPs in hospitals.

#### Exclusion criteria

Not qualitative research or studies with qualitative data that were analyzed using quantitative methods; written in another language than English or Chinese; research not published in peer-reviewed journals, case reports, conference proceedings, poster abstracts, and theses. In addition, while we excluded systematic reviews and other reviews, we reviewed their references to identify possible relevant studies.

### Search outcomes

**Figure 2:**
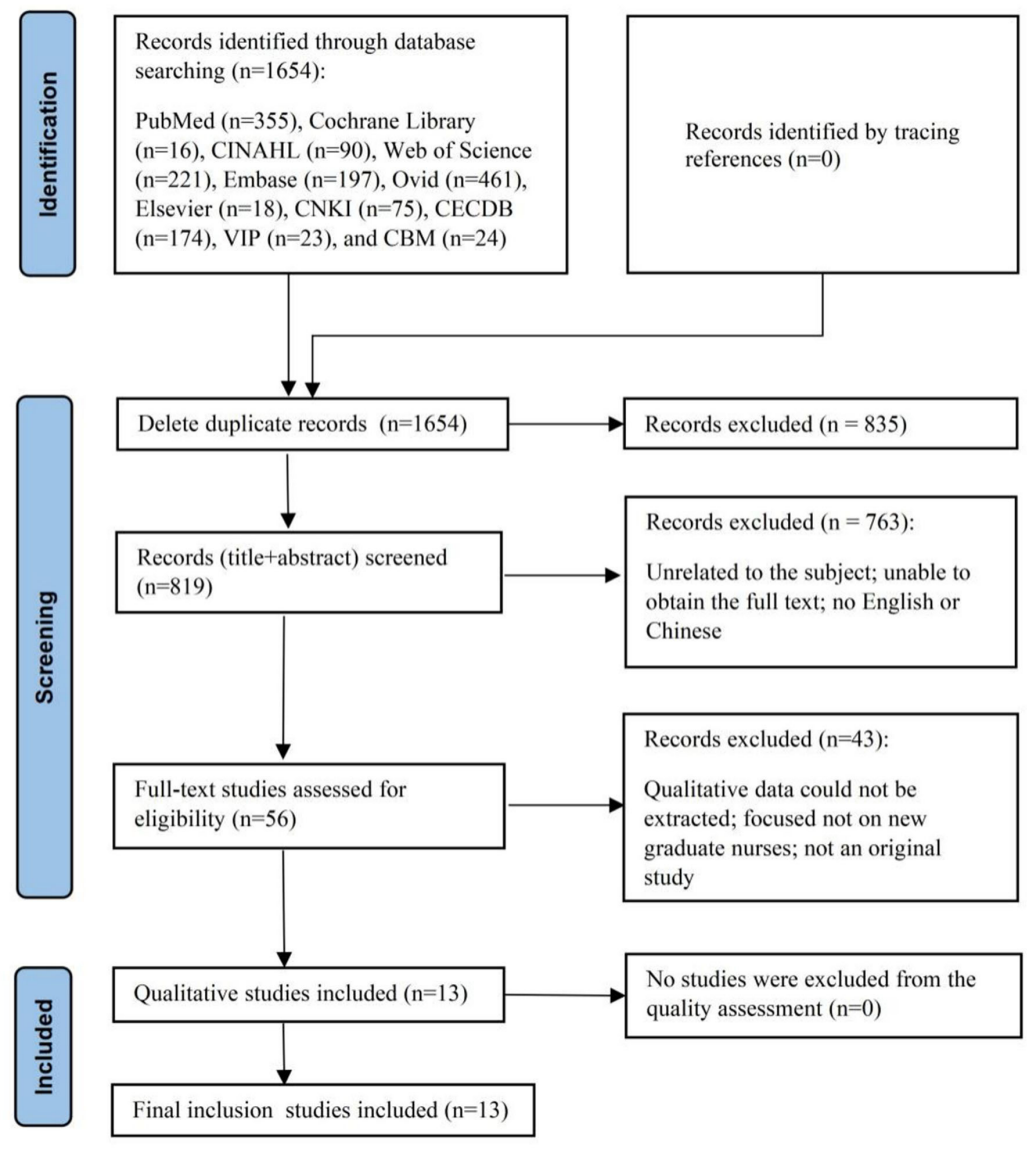
PRISMA flowchart.

Two researchers independently screened and extracted the literature according to the inclusion and exclusion criteria. An initial search using the above strategy yielded a total of 1,654 articles. First, the titles and abstracts of the articles were read to exclude those unrelated to the subject, were repetitive, and full text could not be obtained. Subsequently, 1,603 articles were excluded. After reading the full texts, 43 articles were excluded, and finally, 13 articles were identified as relevant. Also, no articles were traced from references. This search process is illustrated in Figure 2.

### Quality appraisal

Two researchers independently assessed the methodological quality of the 12 included studies. Initially, the authors worked independently using the Joanna Briggs Critical Assessment Tool for Methodological Quality Assessment (23). This evaluation tool is widely applicable in the appraisal of qualitative research. The quality appraisal of research mainly focuses on the internal authenticity of the research, that is, the degree to which the research results are close to the true value, in order to determine whether there is bias. The evaluation tool consists of 10 questions designed to quickly and efficiently evaluate the studies with a simple yes, no, or unclear to each question. Each criterion was allocated a score (Yes = 2, No = 0, Unclear = 1), giving a total score of 20 for each study. These scores were then converted to a percentage. Subsequently, the results were discussed to reach a consensus, studies with a score of more than 70% were considered acceptable after quality appraisal, otherwise they were excluded.

### Data extraction

A comprehensive study was conducted to characterize the quality of the content and assess the methodological development in the collected studies (24). The extracted data included the author, the year of publication, country or region, research method, research subjects, interesting phenomena, and main research results. The results were cross-reviewed by two investigators, and any disagreement was resolved by discussion with a third investigator.

### Data analysis and synthesis

We used meta-aggregation to synthesize the findings of the qualitative studies (25). This method of systematic review involves categorizing and re-categorizing the synthesized findings of two or more studies. First, each selected article was read several times to increase the understanding of research objectives, methods, and conclusions. Next, the results of each study are extracted, along with the research results showing that the results of data and text. The consistency between the research results and supporting data was assessed by two researchers independently. Each finding provided its own level of credibility (23). For qualitative data, there are three levels of credibility: (1) Unequivocal (U): relates to evidence beyond a reasonable doubt, which may include findings that are matter of fact, directly reported/observed and not open to challenge; (2) Credible (C): those that are, albeit interpretations, plausible in light of data and the theoretical framework. They can be logically inferred from the data; (3) Not Supported (NS): when 1 nor 2 apply and when most notable findings are not supported by the data. Results were then encoded according to their meaning and content. Researchers looked for similarities and differences between the findings and the textual data, and the meanings of the original data set were classified. For each theme, when needed, sub-themes were also developed following the same process. Finally, these categories were repeatedly assessed to identify the similarities and obtain synthesized results.

### Ethical considerations

This meta-analysis was carried out in compliance with the Preferred Reporting Items for Systematic reviews and Meta-Analyses (PRISMA) guidelines and referred only to published data. Therefore, no ethical approval was required. However, the ethical approval in the included empirical qualitative studies was assessed.

## Results

The 13 studies (26–38) were conducted in the following countries: China (*n* = 8), USA (*n* = 2), Iran (*n* = 1), Nepal (*n* = 1), and Chinese Taiwan (*n* = 1). These studies involved 189 new nurses. Research methods included: phenomenological approaches (*n* = 11), descriptive qualitative analyses (*n* = 1) and grounded theory (*n* = 1). All studies were published after 2012 and were original articles. The results of the literature quality appraisal were shown in Table 1, the extraction results were presented in Table 2. The PRISMA was used as a basis for the results of syntheses. Three major themes emerged from the selected studies, reflecting the real experience of new nurses in experiencing and adjusting to stressful events in NRPs. These themes were: multi-dimensional stressors, somatic and emotional responses, coping resources and coping methods. The themes were divided into several sub-themes of meaningful units, as demonstrated in Table 3.

**Table 1:** Quality assessment of included studies in accordance.

References*	Q 1**	**2**	**3**	**4**	**5**	**6**	**7**	**8**	**9**	**10**	**Result (%)**
Thomas et al. (26)	Y	Y	Y	Y	Y	N	N	Y	Y	Y	16/20 (80%)
Shrestha and Joshi (27)	Y	Y	Y	Y	Y	N	Y	Y	Y	Y	18/20 (90%)
Zamanzadeh et al. (28)	Y	Y	Y	Y	Y	Y	Y	Y	Y	Y	20/20 (100%)
Liang et al. (29)	Y	Y	Y	Y	Y	N	N	Y	Y	Y	16/20 (80%)
Hu et al. (30)	Y	Y	Y	Y	Y	N	N	Y	Y	Y	16/20 (80%)
Dai et al. (31)	Y	Y	Y	Y	Y	N	N	Y	Y	Y	16/20 (80%)
Yang et al. (32)	Y	Y	Y	Y	Y	N	N	Y	Y	Y	16/20 (80%)
Liang et al. (33)	Y	Y	Y	Y	Y	N	N	Y	Y	Y	16/20 (80%)
Tang et al. (34)	Y	Y	Y	Y	Y	N	N	Y	Y	Y	16/20 (80%)
Ouyang et al. (35)	Y	Y	Y	Y	Y	N	N	Y	Y	Y	16/20 (80%)
Jiang et al. (36)	Y	Y	Y	Y	Y	N	N	Y	Y	Y	16/20 (80%)
Urban and Barnes (37)	Y	Y	Y	Y	Y	Y	Y	Y	Y	Y	20/20 (100%)
Tan et al. (38)	Y	Y	Y	Y	Y	N	N	Y	Y	Y	16/20 (80%)

*Critical appraisal (n = 10) of (Y = yes; N = no; U = unclear; NA = not applicable).

**Question = Q.

**Table 2:** Key features and characteristics of 13 included studies.

**References**	**Country**	**Research method**	**Participants**	**Aim**	**Results**
Thomas et al. (26)	USA	Phenomenological approach; semi-structured interviews	11 new registered nurses in acute care hospital environments	To understand the transition experience of new registered nurses during the first year of clinical practice	Four themes: feelings of frustration and being overwhelmed, ongoing support of preceptors, identified fears, ongoing feedback during orientation
Shrestha and Joshi (27)	Nepal	Phenomenological approach; semi-structured interviews	6 new staff nurses in a hospital in Kathmandu	To explore the sources of stress during the transition of newly graduated nurses into clinical practice	Four themes: stressful initial days, leaving the nest, supporting work environment, hierarchical work pattern
Zamanzadeh et al. (28)	Iran	Phenomenological approach; semi-structured interviews	12 nurses with less than a year's experience from various wards in hospitals in Iran	To investigate nurses' preparation for independent professional practice and nurses' transition from college to professional practice	Three themes: poor professional efficiency, lack of self-assurance, unhealthy emotional reactions
Liang et al. (29)	China	Phenomenological approach; semi-structured interviews	12 nurses in traditional Chinese medicine hospital who have participated in standardized training in Chongqing	To understand the main pressure of standardized training of nurses in traditional Chinese medicine hospitals	Four themes: the nature of the job, the lack of professional knowledge, the frequent examination or study, complicated interpersonal relationships
Hu et al. (30)	China	Grounded theory; field observation; semi-structured interviews	25 newly graduated pediatric nurses in Shanghai	To identify stressors of newly graduated pediatric nurses at a children's hospital in Shanghai, China	Five themes: low work status, insufficient professional competence, heavy workload, inadequate supportive systems, and uncertainty of career development.
Dai et al. (31)	China	Phenomenological approach; semi-structured interviews	15 new nurses in a hospital in Hubei Province, China	To understand the problems and real feelings of new nurses in clinical practice	Six themes: lack of sense of belonging, lack of experience, lack of specialized knowledge reserve, learning pressure, lack of communication skills, sense of being needed
Yang et al. (32)	China	Phenomenological approach; semi-structured interviews	20 new nurses in the respiratory department of a hospital in Fujian Province, China	To understand the work pressure of new nurses in the respiratory department and discuss the measures to reduce the pressure	Five themes: pre-employment fear, nursing, and rescue of severe patients cause stress, diversified forms of pressure, way to relieve the pressure is single, lack of leadership and family support
Liang et al. (33)	Chinese Taiwan	Descriptive phenomenological approach; semi-structured interviews	25 NGNs working in clinical settings in Taiwan	To discover Taiwanese Newly graduated nurses' (NGNs) experiences of work challenges	Four themes: being tense as if walking on thin ice, suffering physical exhaustion and mental stress, entering and adjusting to the profession, gaining more confidence
Tang et al. (34)	China	Phenomenological approach; semi-structured interviews	10 newly graduated nurses who participated in standardized training in Sichuan Province, China	To explore the psychological experience of nurses during standardized training and provide more effective and scientific teaching basis for clinical nursing teaching	Five themes: interpersonal communication pressure, lack of awareness of self-protection, gap between reality and ideal, monotonous teaching activities, lack of nursing skills
Ouyang et al. (35)	China	Phenomenological approach; semi-structured interviews	18 nurses who have completed 2 years of standardized training	To discusses the psychological stress of new nurses in training and to find the advantages and disadvantages of training	Five themes: psychological changes, stressors, social support, gains, needs
Jiang et al. (36)	China	Phenomenological approach; semi-structured interviews	8 operating room nurses in a hospital who finished standardized training	To analyze the real experience of operating room nurses in the standardized training	Three themes: stress and coping, confusion and lack of belonging, embarrassment and self-adjustment
Urban and Barnes (37)	USA	Phenomenological approach; semi-structured interviews	15 new graduate nurses in an acute care settings in the southwestern United States	To describe the lived experience of Newly graduated nurses (NGNs) during this period of early independent practice	Three themes: feeling overwhelmed, navigating work-based relationships, finding their flow
Tan et al. (38)	China	Phenomenological approach; semi-structured interviews	12 new nurses in a hospital in Fuzhou	To understand the pressure changes of new nurses in different periods of clinical work and their adaptation, so as to help new nurses better adjust their pressure	Two themes and 10 secondary theme: 3 months on the job (unclear work responsibilities, unfamiliar environment, interpersonal relationship, insufficient clinical knowledge and skills, low salary, many assessments); 6 months on the job (heavy workload, important independent duty, difficult nurse-patient relationship, tense relationship with colleagues)

**Table 3:** Thematic synthesis findings.

**Descriptive themes**	**Sub-themes**
Multi-dimensional stressors	Lack of competence for the position Unfamiliar working environment Poor interpersonal communication Study and exam pressure Poor social status and treatment
Somatic and emotional responses	Physical fatigue Tension and fear Anxiety and depression Experience of loneliness
Coping resources and coping methods	Self-regulation and adaptation Taking action to improve capabilities Positive self-awareness Support from multiple sources

### Theme 1: Multi-dimensional stressors

#### Lack of competence for the position

Most new nurses enter clinical practice immediately after graduating from university. At the beginning of their careers, they had a significant lack of clinical knowledge and skills and indicated that they were not prepared for clinical responsibilities. The new nurses complained of the gap in their competence. “*For the first five or six months, I was totally confused. We had to provide a variety of care in the ward, and I realized that my college training was irrelevant. I was puzzled by how expansive the field was, and I lacked many of the required skills*” (27); “*I don't know much about hospital nursing, and practice at schools and hospitals is totally different*” (30).

#### Unfamiliar working environment

When entering a new work team, new nurses often felt awkward and disheartened because they were not familiar with the surrounding environment and personnel, which represented a source of pressure that could not be ignored. A nurse reported her annoyance at unfamiliar surroundings, which made her work less productive. “*It took some effort to adapt to the new environment of the department. At the beginning, I had to review the position of things by myself every day, fearing I would not be able to find drugs during the rescue intervention*” (38). At the same time, due to frequent rotations, new nurses are repeatedly in a cycle of adaptation—departure—re-adaptation, lacking a sense of belonging and security. Also, the process of adaptation to the new environment is stressful. “*When you go to a new department, you are unfamiliar with people working there and feel more pressure*” (34).

#### Poor interpersonal communication

During NRPs, it is important for new nurses to communicate with superiors, tutors, colleagues, patients, etc. Good and effective communication is a powerful incentive to perform the work well. Some newly graduated nurses reported that they were in the groping stage of relationship establishment and lacked of communication skills and ability due to their poor experience in the new job (31, 32, 38). These are their statements about communication with colleagues or patients: “*Sometimes I want to communicate with the doctor, but I don't know exact terminology*” (31); “*If* a *patient was in a bad mood, I was afraid of saying the wrong thing in order not to lose the patients' trust*” (31).

#### Study and exam pressure

In order to meet the training needs, new nurses during NRPs have to undergo many professional examinations and ability assessments, all of which require frequent learning. To meet the requirements, they even need to study and train on their own during breaks from work. Many new nurses saw this as a major cause of stress. “*Each time during department inspection, I would be asked questions by the head nurse. It was impossible to avoid them*” (31). They complained about the frequent examinations: “*From theory to operation, from western medicine to Chinese medicine, every day is not a test, but preparing for the test*” (29).

#### Poor social status and treatment

Some newly graduated nurses expressed that the social contempt and belittling of the nursing profession brought a psychological burden to them. Although these rumors were not true, it was irritating to new nurses. “*My parents were unhappy with my career choice. In their opinion, I could get any job, and any job was better than being a nurse, which they compared with being a servant. In addition, they felt ashamed of my work*” (30). Some new nurses expressed dissatisfaction with their salary level, saying that their workload was not proportional to their income. Low income made life more difficult, and the low level of job satisfaction during NRPs was not conducive to career development. “*I work very hard, but my salary is very low, not enough for rent and basic living expenses*” (38).

### Theme 2: Somatic and emotional responses

#### Physical fatigue

Overall, we found that most studies reported various physical conditions among participants (27, 29, 30, 32, 33, 35, 36, 38), including, but not limited to, sleep disturbances, headaches, endocrine function disorder, and breathlessness. These conditions could be attributed to long working hours, high working intensity and low efficiency. Night shifts were especially difficult to accommodate. “*There are always quite a few night shifts... I could not bear such intensive work all the time. My face would break out*” (30); “*I still could not adjust to working in shifts. I would feel exhausted while working the night shift, and then I could not sleep as I was too exhausted*” (33). Some new nurses often had to work overtime to complete all the tasks. One nurse expressed her feelings about this as follows: “*Working an additional shift was terrible. However, sometimes I was informed to work another 12 hours the next day. In fact, such consecutive night shifts were overwhelming*” (30).

#### Tension and fear

For new graduate nurses who had just stepped into their jobs and had not yet established a mature professional level, tension was inevitable. New nurses feared that they would not be able to perform specialized nursing work alone or make mistakes in unfamiliar or difficult work during NRPs. “*I'm afraid of having to work with those obese patients who need to have their blood drawn for arterial blood gas analysis, which is a great challenge for me. Also, there are a lot of medicines I am not familiar with, I'm afraid of sending the wrong medicine*” (32). In daily work, many patients were not friendly to novices. New nurses might feel nervous in the face of distrust from patients or their family members. One participant recalled: “*Some parents were not very nice. They would threaten us when we were giving an intravenous injection. It was terrible*” (30).

#### Anxiety and depression

Work pressure caused nurses to suffer from anxiety and even lose confidence in themselves. If they couldn't adapt well, it would soon affect their physical and mental health. “*I felt a little nervous. I was the most afraid to see the department phone number, as it meant that I did something wrong*” (32). In the face of complex work problems, they discovered that they did not have sufficient knowledge and skills to cope with the problem. They were unable to cope with high frustration and developed severe self-doubt and self-blame. One nurse recalled, “*I would cry and despair when I would get home, and say to myself, ‘I cannot do this.' Yeah, it is stressful. It would make me ask myself questions like, ‘Is this the right area for me?' and ‘am I doing the right thing?'*” (37).

#### Experience loneliness

When a nurse starts a professional career, he or she encounters a completely different environment from the one experienced at the college. It is easy to experience loneliness without the support of one's coworkers and managers, which in turn further intensifies stress. One participant remarked the following: “*When I started my job, there were many things that I didn't know. I would ask my coworkers for help, but they were busy and wouldn't help much. I felt quite lonely; my coworkers treated me like an outsider*” (27). During NRPs, nurses might feel lonely and find it hard to fit in when they are in departments different from their own. Another participant remarked, “*They always saw you as someone who would eventually leave, you're not from this department. I really wanted to fit in, but I couldn't*” (36).

### Theme 3: Coping resources and coping methods

#### Self-regulation and adaptation

New graduate nurses often adjusted their emotions and relieved work pressure through rest, sports, or various entertainment. “*(I often) play ball games with friends, or do some exercise, so as to improve my mood*” (38). At work, the new nurses continued to grow professionally, gradually improving their ability to regulate emotions and more rationally deal with setbacks. One participant saw it as a process of change, “*I found I was not as angry as before when I would encounter the same situation. For instance, if patients asked me the same question three times or more, I would get impatient and angry. Now, I am not as impulsive, and I do not get angry. I feel more confident because of my good emotional control*” (33).

#### Take action to improve capabilities

Taking positive actions to gain more knowledge and improve work capabilities is what new nurses need to do to adapt to the post. Some new nurses had correct self-assessment, through independent knowledge learning and skill practicing, to improve their weaknesses. “*I knew my IV skills and other skills were not good. I did lots of homework in the skills training room after work to make up for it*” (29). They gained confidence when they were able to easily solve problems in their patient care practice. “*When patients ring me in diverse situations, such as IV lock or BP drop, I could solve their problems and handle them very well, thus gaining confidence from these experiences*” (29).

#### Positive self-awareness

During the NRPs, new nurses gained more comprehensive clinical nursing ability, broader vision, and clearer self-recognition. The enhancement of professional confidence helped them overcome setbacks and advance their future career. “*After the training, I felt that my strength in all aspects has been enhanced. I recalled the shortcomings during my previous nursing work and knew how to improve them in the future. After many experiences, I felt that this career path was valuable, and I was inspired to work harder in the future without considering changing my career*” (35).

#### Support from multiple sources

External support from nursing managers, colleagues, friends, and families could effectively help nurses improve their ability to adapt to stress and psychological adjustment. Many nurses expressed that they received good organizational support that was helpful to their work (26–29, 35, 38). “*I found a welcoming atmosphere in the work setting*—*an atmosphere which I appreciated. I was encouraged to ask questions. I found nursing in charge and coworkers being good at giving feedback as well*” (27). Participants reported that professional tutors had an important role in the transition experience. “*I had had so much support when I was working. My preceptor answered all of my questions*” (26). Nurses expressed that they needed care and support from other people outside, such as their families and friends, which could give them a great spiritual boost during tough times. “*I would chat with friends to decompress; friends would comfort and enlighten me, thus reducing the psychological burden*” (38). However, some new nurses reported dissatisfaction with organizational support during NRPs. “*My preceptor was very busy, and rarely communicated with me. He was also frequently substituted*” (31).

## Discussion

This systematic review of 13 qualitative studies on the stress experience and coping styles of new nurses during NRPs, followed by a meta-synthesis, was performed on various databases after a manual search. The meta-synthesis process of this study was rigorous, and the results were reliable, thus can be used as the application basis for evidence-based practice. The main findings discussed in this section indicated that nurses faced a lot of physical and emotional stress during Nurse Residency Programs. These stressors came from lack of professional ability, inadequate professional preparation, work and social environment, and similar. New nurses found reasonable ways to cope with stress at the beginning of their careers. They were trained to improve clinical competence and self-efficacy, while external support from nursing managers, colleagues, friends, and families had an essential role. However, more strategies are needed to enhance this effect, especially in NRPs.

The stress among nursing staff is a global problem, especially for the nurses with low seniority, who are generally regarded as the group with high pressure and high turnover rate (39). Previous studies showed that nearly half of new nurses experienced severe symptoms of illness associated with very high-stress levels (40). It has been reported that work overload and extended working hours, difficulties in nurse-patient communication, high occupational risk, and failure to realize occupational value are common sources of stress among new nurses at work (41–44). In addition, they judged that negative emotions such as anxiety, loneliness, and emotional vulnerability were common among new nurses, which is consistent with our integrated results (45, 46). The high-stress state and negative emotions reduce the professional experience of new nurses, which poses a threat to patients' safety and nursing quality, and is not conducive to the retention of nurses, social recognition of this profession will also decrease, creating a vicious circle (15, 47). Therefore, targeted and effective changes should be made in the NRPs.

In the face of unfamiliar work content, working environment, and colleagues, this sense of inadaptability will lead to the lack of organizational identity of new nurses in practice and even directly affect the professional values, work engagement, the sense of belonging to the organization and the competence of the post (48, 49). The person-organization fit theory studies the adaptability between individuals and the environment and reflects the interaction between behavior subjects and the environment, emphasizing the consistency or complementarity between employees and the organizational environment (50). It is necessary to pay attention to the adaptation and matching of employees' knowledge and abilities with specific job demands and also to pay more attention to the conformity of employees' internal characteristics with the potential needs of the organization (51). Therefore, the connotation of this theory can be fully incorporated in the goal-setting of NRPs, where the individual needs of new nurses should be taken as the first priority, and the pressure of role adaptation should be reduced. For example, more emphasis should be placed on welcoming and accepting new employees in the department, the gradual and guiding nature of work content, and the scientific and reasonable assessment methods. Greater focus should also be placed on the nurse's inner recognition of the role and their actual ability to improve.

The nursing occupation is characterized by high work intensity and heavy workload, so the health of new nurses is often affected. Sleep disorders, headaches, musculoskeletal disorders, endocrine dysfunction, and other symptoms have also been reported in past studies to frequently appear in clinical work, affecting nurses' health (52–54). As newly graduated nurses are not familiar with the work content, they need more time to adapt. Meanwhile, during NRPs, they are also required to attend to more additional theoretical training and exams, which requires an investment of more of their personal time. High stress and lack of rest are very bad for their health. In recent years, Objective Structured Clinical Examination (OSCE) (55) and Mini-Clinical Evaluation Exercise (Mini-CEX) (56) have been proven to be very suitable for nursing Clinical competence evaluation. These efficient models should be considered for the evaluation of new nurses.

Gillespie et al. (57) speculated that positive coping benefits nurses' development, which is consistent with our findings. A positive coping style alleviates negative emotions by improving the individuals' understanding of negative events and enhancing the ability to solve and cope with problems. When confronted with difficulties at work, some nurses can resolve the issues in a short time through positive physical actions, which often depend on their self-efficacy and execution. This ability helps them to feel more confident in finding solutions (58). A proactive problem-solving approach is a good guide in a clinical work environment. Nurse leaders should focus on the cultivation of team action and problem-solving character. Professional competence and knowledge are at the basis of how nurses deal with difficult situations, which also directly affects the quality of hospital care and patient satisfaction.

Resilience implies the ability to bounce back or recover easily when confronted by adversity, trauma, misfortune, or change (59). The key point of resilience is to adapt to various environments. Resilience in nursing was defined by the researchers as a measure of a nurse's ability to cope with stressors and mental health threats, where resilient people were emotionally calmer while dealing with catastrophic situations (60). For nurses, resilience is one of the factors that reduce their stress level and increases endurance. However, nurses with fewer working years tend to feel challenged or even escape when facing pressure due to the lack of experience and skills, which negatively affects their resilience (61). Therefore, in NRPs, prompt attention should be paid to the resilience training of inexperienced new nurses.

Also, the need for self-actualization is a common human need, which stimulates a positive attitude and willingness to improve professional qualities. Thus, cultivating a positive personality ensures that individuals acquire healthy professional experience (62). As reported by Jnah and Robinson (63), the positive emotions and self-efficacy had a positive effect on the resilience, indicating a high degree of confidence in the face of difficulties. Positive psychological strength and excellent psychological qualities improve adaptability (64). For a new nurses, positive professional identity and self-cognition are favorable factors for their career development. Correct professional values underline clinical nursing practice, their overall quality, and the improvement of the quality of nursing service. Therefore, training to reduce emotional exhaustion and improve the personal sense of achievement should be strengthened. A positive, purposeful rumination is considered worth cultivating. Deliberate rumination refers to the adaptive cognitive process of paying attention to the negative emotions or experiences caused by traumatic events, actively, consciously, and purposefully explaining traumatic events, seeking meaning, and exploring inner feelings (65), which is beneficial for new nurses to improve their resilience and post-traumatic growth (66). Similarly, targeted occupational psychoeducation for new graduate nurses before they take up a job should be paid attention to. In the university curriculum, courses of professional values need to play a role in providing them with more confidence.

Social support refers to the social resources provided by formal or informal support groups that are perceived subjectively and/or received objectively by individuals (67). During NRPs, nursing managers should focus on establishing a support system and creating a positive work atmosphere, which can help new nurses improve their work engagement and better integrate into the team (68). More scientific and humanized nursing management like Management by Walking Around (MBWA) should be advocated. This is a mode in which managers take the grassroots approach, timely find and solve problems, and provide practical and effective help to employees to improve each department's overall efficiency. In essence, it is a kind of harmonious informal communication whose ultimate goal is to serve patients and society (48). For leaders, the reasonable demands of new nurses should be given full attention. In addition, negative psychological situations caused by excessive workload or unfair treatment should be avoided. They should make a reasonable human resource plan and establish a scientific working process according to workload and personnel allocation ratio (69). Research showed that the tutorial system was effective in establishing organizational support, and it was an educational management model that respected individual differences and adapted to learning objectives (70). Starting from the needs of new nurses, clinical tutors should provide one-to-one help with their work, study, and life and provide them with maximum professional and spiritual support. At the same time, establishing a good teacher-student relationship foundation can increase the connection between new nurses, colleagues, and patients and improve personal belongingness and psychosomatic adaptability.

Our review identified the embodiment and causes of physical and mental stress faced by new nurses during NRPs, and how it affected their health. The results provide a valuable basis for future research. The innovation lies in summarizing how new graduate nurses cope with stress at work from both positive and negative aspects, and the corresponding coping effects under different situations. Then, we highlighted the role of organizational and social relationships in coping with stress in NRPs, in addition to individual abilities. This would make it clear that the relevance of the human factor in explaining nurses' physical and mental experience of career transitions. In addition, we emphasized that coping with difficulties and stress and eliminating negative emotions is a process change. The coping ability and resilience of individual changes from weak to strong with time and experience, which will require managers to explore how to establish strategies from the practice of NRPs, to help new nurses adapt and grow quickly in their new roles.

The present study has several limitations. First, according to the inclusion criteria for literature, only qualitative and mixed-method studies published in indexed journals, written in English or Chinese, were selected. Therefore, gray literature and some papers or dissertations were not searched, which may lead to information bias. In addition, due to cultural and policy differences, the definition and specific content of NRPs in different countries and regions are not completely the same, different researchers have their understanding and interest, at the same time, nurses' stressors, social relations and professional values are greatly affected by different regional cultures, which may cause differences in results.

New nurses face a lot of physical and emotional stress during NRPs, which negatively impacts their physical and mental health. This directly affects the retention rate of new nurses, and it is difficult to ensure the quality of clinical care and patient safety. NRPs are a critical period for the professional career growth of new nurses. Correctly guiding their career transition is a difficult but significant task. Future in-depth research should focus on how to improve nurses' stress coping ability and enhance their sense of professional value. Educational institutions should explore the change of teaching content, hospital management should implement effective management strategies, improve the ability of nurses and establish organizational support. Researchers and nursing managers need to make the design and implementation of NRPs strategy more progressive, ensure the career growth of new nurses, maintain the enthusiasm and sustainability of the nursing team, and achieve the goal of improving the quality of nursing.

## Data availability statement

The original contributions presented in the study are included in the article/supplementary material, further inquiries can be directed to the corresponding authors.

## Author contributions

PH: conceptualization, methodology, formal analysis, writing—original draft, and writing—review and editing. XD: conceptualization, methodology, writing—original draft, and writing—review and editing. LW: conceptualization, methodology, formal analysis, and writing—review and editing. XZ and JJ: methodology, formal analysis, and writing—review and editing. All authors contributed to the article and approved the submitted version.

## Funding

This work was supported by Shanghai Shenkang Hospital Development Center Clinical Science and Technology Innovation Project (SHDC12021611) and Shanghai Medical Union Theory Research Key Project (2022YGL10).

## Conflict of interest

The authors declare that the research was conducted in the absence of any commercial or financial relationships that could be construed as a potential conflict of interest.

## Publisher's note

All claims expressed in this article are solely those of the authors and do not necessarily represent those of their affiliated organizations, or those of the publisher, the editors and the reviewers. Any product that may be evaluated in this article, or claim that may be made by its manufacturer, is not guaranteed or endorsed by the publisher.
